# A 20-bp insertion/deletion (indel) polymorphism within the *CDC25A* gene and its associations with growth traits in goat

**DOI:** 10.5194/aab-62-353-2019

**Published:** 2019-06-17

**Authors:** Wenbo Cui, Nuan Liu, Xuelian Zhang, Yanghai Zhang, Lei Qu, Hailong Yan, Xianyong Lan, Wuzi Dong, Chuanying Pan

**Affiliations:** 1 College of Animal Science and Technology, Northwest A&F University, Key Laboratory of Animal Genetics, Breeding and Reproduction of Shaanxi Province, Yangling, Shaanxi, China; 2 Shaanxi Provincial Engineering and Technology Research Center of Cashmere Goats, Yulin University, Yulin, China; 3 Life Science Research Center, Yulin University, Yulin, China; 4 College of Animals Science and Technology, Northwest A&F University, No. 22 Xinong Road, Yangling, Shaanxi 712100, China

## Abstract

Cell division cycle 25A (*CDC25A*), a member of the *CDC25* family of phosphatases, is required for progression from G1 to the S phase of the cell cycle. *CDC25A* provides an essential function during early embryonic development in mice, suggesting that it plays an important role in growth and development. In this study, we used mathematical expectation (ME) methods to identify a 20-bp insertion/deletion (indel) polymorphism of *CDC25A* gene in Shaanbei White Cashmere (SBWC) goats. We also investigated the association between this 20-bp indel and growth-related traits in SBWC goats. Association results showed that the indel was related to growth traits (height at hip cross, cannon circumference, and cannon circumference index) in SBWC goats. The height at hip cross of individuals with insertion/insertion (II) genotype was higher than those with insertion/deletion (ID) genotype (P=0.02); on the contrary, the cannon circumference and cannon circumference index of individuals with ID genotype were superior when compared with those with II genotype (P=0.017 and P=0.009). These findings suggest that the 20-bp indel in the *CDC25A* gene significantly affects growth-related traits, and could be utilized as a candidate marker for marker-assisted selection (MAS) in the cashmere goat industry.

## 1 Introduction

Goat is one of the most important livestock species and the oldest economic domesticated species, being used for meat, milk, and cashmere all over the world. Also, goat is characterized by its high reproduction rate, high quality of meat, strong compliance, and easy management, and is widely cultivated on a global scale, especially in China, India, and Pakistan (Liu and Zhou., 2015). With the improvement of society, goat meat is gradually consumed by the masses because of its high protein, low fat, and low cholesterol (Mao et al., 2012). Shaanbei White Cashmere (SBWC) goat is a cashmere and meat type of goat. Because of the high quality of meat, SBWC goats have been the main economic variety in Yulin city, Shaanxi province. In order to improve the comprehensive economic benefits of SBWC goats, it is necessary to study the growth and development traits so that we are able to lay a foundation for vigorously developing the meat performance of cashmere goats.

Goat production relies mainly on grazing on communal lands that hardly provide the minimum nutrient requirements due to overstocking and degradation in Asia, Africa, and Latin America. Appropriate breeding strategies should be designed to promote conservation and improvement of goat unique attributes (Escareño et al., 2013). The molecular marker-assisted selection (MAS), which is commonly used in molecular breeding, has become an important part of modern breeding technology systems. The principle of the technology is to use the molecular markers or functional markers closely linked to the target gene to accurately identify the genotypes of different individuals in the hybrid progeny, and to carry out the breeding technique based on the assisted selection (Bai et al., 2018). At present, MAS based on relevant genetic variants is used extensively to improve traits with low heritability, such as those associated with growth and reproduction (Silpa et al., 2018; Zhang et al., 2018). Single-nucleotide polymorphism (SNP), insertion/deletion (indel) and structural variation (SV) are the major genetic variations (Mullaney et al., 2010) and the main kinds of MAS. It was reported that a multiallelic indel in the promoter region of the Cyclin-dependent Kinase Inhibitor 3 gene was significantly associated with body weight and carcass traits in chickens (Li et al., 2018). A 10-bp indel polymorphism in the bovine *PAX7* promoter altered the binding of the transcriptional factor *ZNF219* and modulated promoter activity and gene expression in Chinese cattle (Xu et al., 2018). Also, some literature illustrated that both SNP and indel variations were closely related to certain traits, including the growth traits of cashmere goat (Zhang et al., 2015; Wang et al., 2019a). However, major genes affecting the growth traits of goats have not been found, and further research is needed.

Currently, whole-genome sequencing and genome-wide association studies (GWASs) are used to explore genetic variants strongly associated with production traits (Wang et al., 2016; Rahmatalla et al., 2018; Yang et al., 2018). In 2016, a study using whole-genome analysis identified several genes, which may have contributed to the phenotypes in body size in goat populations from eight domesticated goat breeds, as potentially critical for fecundity, including cell division cycle 25A (*CDC25A*) (Wang et al., 2016). *CDC25A* is a member of the *CDC25* family of phosphatases and also a dual-specificity protein phosphatase. Several literature studies have shown that *CDC25A* is one of the most crucial cell cycle regulators which is required for activation of the apoptotic cell cycle pathway (Shen and Huang, 2012; Biswas et al., 2017). In terms of embryos, promoting *CDC25A* expression can regulate cell proliferation and axis extension during gastrulation in zebrafish. *CDC25A* ensured the health and genomic stability of the developing embryo in mice (Lee et al., 2009; Liu et al., 2017). However, the relationship between *CDC25A* and growth traits has never been reported in goat. Therefore, it is essential to explore the association between the *CDC25A* gene and the growth traits of SBWC goats.

In this study, a 20-bp indel polymorphism of the *CDC25A* gene was found in SBWC goat by using mathematical expectation (ME) methods (Yang et al., 2016). Furthermore, this 20-bp indel polymorphism of the *CDC25A *gene was found to be associated with the growth traits of SBWC goats. Our findings provide a basis for further research on the underlying causal mutation and suggest hypotheses for further studies leading to the application of MAS for goat breeding.

## 2 Materials and methods

All experimental procedures were approved by the Review Committee for the Use of Animal Subjects of Northwest A&F University, China. The animal experimentation, including sample collection, was performed in agreement with the guidelines of the ethics commission.

### 2.1 Animals and data collection

All experimental animals were raised at the SBWC breeding farmland and managed under the same conditions. A total of 729 ear tissue samples from female SBWC goats were collected randomly from the SBWC breeding farm in Yulin, Shaanxi province (Wang et al., 2018a, 2019b). Growth traits for all selected unrelated individuals were measured, including body height (BH), body length (BL), heart girth (HG), chest depth (ChD), chest width (ChW), height at hip cross (HHC), and cannon circumference (CC); consequently, body length index (BLI), heart girth index (HGI), chest width index (ChWI), cannon circumference index (CCI), and body trunk index (BTI) were also calculated on the basis of our reported description (Jia et al., 2015; Yang et al., 2017). All tissues were stored at -80 ${}^{\circ }$C until used for analysis and DNA experimentation.

### 2.2 DNA extraction and genomic DNA pools construction

The DNA was extracted using the high-salt extraction and phenol chloroform methods (Lan et al., 2007) and then diluted to a standard concentration (10 ng µL${}^{-1}$) and stored at -20 ${}^{\circ }$C for the detection of genetic variation. The Nanodrop 1000 instrument was used to assess DNA purity ($A_{260/280}$ ratio) and quality.

A total of 50 DNA samples were randomly selected from each breed to construct genomic DNA pools. Genomic DNA samples were diluted to a standard 10 ng µL${}^{-1}$ concentration and individual aliquots of DNA samples were transferred to a single tube to ensure that a constant amount of each DNA sample was transferred to the pool (Yang et al., 2016; Li et al., 2017). The pool was then mixed gently and uniformly. The genomic DNA pools were used as a template for polymerase chain reaction (PCR) amplification and then PCR products were sequenced (Sham et al., 2002).

**Table 1:** Amplification PCR primer sequences of *CDC25A* goat gene.

Name	Primer sequences (from 5' to 3')	Tm	Size	Detection
		(${}^{\circ }$C)	(bp)	
P1	F1: ACACCATACATCCGACCTAACT	Touch-	129	ins/ins (II) =129 bp
	R1: ACCAGAAGTAAGCAATGGAGAA	down		ins/del (ID) =129/109 bp
P2	F2: CTGTAACCCGCCAGCTCCATTG	Touch-	168	NA
	R2: ACACAGGGTTCCCTTTGATGGC	down		

NA = not available.

### 2.3 Primer design and PCR amplification

Based on the goat (*Capra hircus*) gene sequence (GenBank accession no. NC_030829.1) and the Ensembl database (http://www.ensembl.org/index.html?redirect=no, last access: 1 September 2018), two potential indel sites were found in *CDC25A* goat gene and two pairs of primers (P1, P2; Table 1) were designed to amplify genomic DNA pools to explore genetic variation in the *CDC25A* goat gene by the NCBI website (https://www.ncbi.nlm.nih.gov/tools/primer-blast/index.cgi, last access: 1 September 2018). The touch-down (TD) PCR reaction procedure was as follows: initial denaturation for 5 min at 95 ${}^{\circ }$C followed by 18 cycles of denaturation for 30 s at 94 ${}^{\circ }$C; annealing for 30 s at 68 ${}^{\circ }$C (with a decrease of 1 ${}^{\circ }$C per cycle); extension for 30 s at 72 ${}^{\circ }$C; another 23 cycles of 30 s at 94 ${}^{\circ }$C, 30 s at 50 ${}^{\circ }$C, and 2 min at 72 ${}^{\circ }$C; and a final extension for 10 min at 72 ${}^{\circ }$C with subsequent cooling to 4 ${}^{\circ }$C (Yang et al., 2016; Kang et al., 2019). The PCR was performed in a 25 µL reaction volume containing 12.5 µL 2× *Taq* Master mix, 0.5 µL of each primer, 2 µL 10 ng µL${}^{-1}$ genomic DNA and 9.5 µL ${\mathrm{ddH}}_{2}O$ (double-distilled H${}_{2}$O).

### 2.4 Indel identification and sequencing

According to previous reports, based on the low frequencies of the 20-bp indel within the *CDC25A* gene and the sample sizes, we designed the most efficient pooling strategy to detect all the individuals by using the ME method (Yang et al., 2016). The PCR products specificity was confirmed by sequencing (Nakamura et al., 2007). The 20-bp indel of *CDC25A* was detected in SBWC breeds by electrophoresis using 3.5 % agarose gel stained with ethidium bromide.

### 2.5 Statistical analysis

Genotypic and allelic frequencies were calculated directly. The $\chi^{2}$ test was carried out to test whether the polymorphism is in Hardy–Weinberg equilibrium (HWE). Polymorphism information content (PIC) was calculated by Nei's method implemented in the GDIcall Online Calculator (http://www.msrcall.com/Gdicall.aspx, last access: 1 December 2018) (Cui et al., 2018). Difference distributions of genotypic and allelic frequencies were analyzed using the $\chi^{2}$ test or Fisher exact tests (when the minimum theoretical frequency was less than 5) in SPSS (version 19.0). For growth traits, analysis of variance was applied to the general linear model and the reduced linear model was as follows: $Y_{ijk}=\mu +\alpha_{i}+\beta_{j}+\varepsilon_{ijk}$, where $Y_{ijk}$ is the observation of the growth trait (body height, etc.) evaluated on the ith level of the fixed factor age ($\alpha_{i}$) and the jth level of the fixed factor genotype ($\beta_{j}$), μ is the overall mean for each trait, and $\varepsilon_{ijk}$ is the random error for the i, j, and kth individual (Wang et al., 2017). Further analysis was performed with SPSS 19.0 software using t test and Mann–Whitney U test; the data were rejected when n<5; P<0.05 was considered statistically significant.

## 3 Results

### 3.1 PCR amplification and sequencing of the 20-bp indel variants of the *CDC25A* goat gene

**Figure 1:**
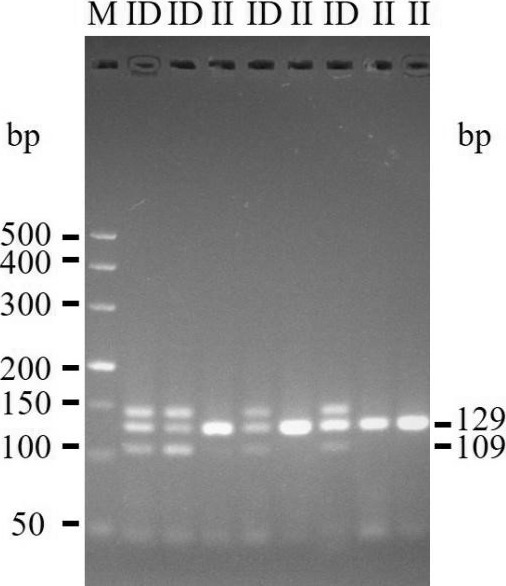
Genotyping of the 20-bp indel determined by PCR amplification product size (3.5 % agarose gel) using P1 primer.

**Figure 2:**
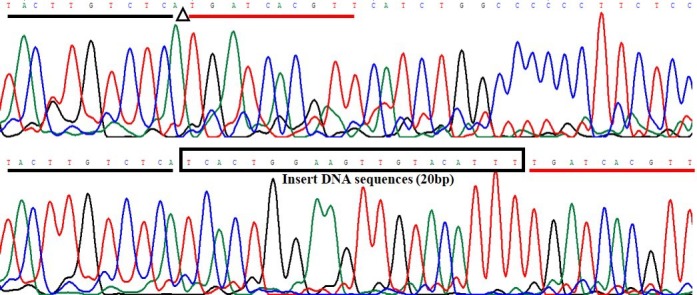
Sequencing maps of 20-bp indel in *CDC25A* goat gene.

The 20-bp indel of the *CDC25A* goat gene was identified by P1 (Table 1) and genotyped by the 3.5 % agarose gel electrophoresis. However, the indel was not identified by P2 (Table 1). For the 20-bp indel locus, only genotypes insertion/insertion (II) and insertion/deletion (ID) were detected: one band (129 bp) for genotype II, and three bands (129/109 bp) and another homoduplex for genotype ID (Fig. 1). Herein, a 20-bp indel in the ninth intron of *CDC25A* goat gene was confirmed (NC_030829.1:g.51746323-51746342delTCACTGGAAGTTGTACATTT). The result of contrasting and analyzing the sequence by software (Bioedit, UK) showed that the indel sequence was “TCACTGGAAGTTGTACATTT” (Fig. 2).

### 3.2 Individuals genotyping by ME method

The low frequency of the 20-bp indel was confirmed in *CDC25A* gene in SBWC goat. Using the ME method, individuals were assigned by order in groups (the least allowed number in a single group) to mixed groups. Dependent on whether there was one single band (129 bp or 109 bp) in the mixed groups of SBWC goat, we needed to detect the genotype. Simultaneously, the number of PCR reactions was decreased. Results showed that the allelic frequencies of I and D were 0.949 and 0.051, respectively. Also, this indel locus was not in HWE and low polymorphic with a polymorphism information content (PIC) (Table 2).

**Table 2:** Allelic and genotypic frequencies and genetic diversity of the 20-bp indel of *CDC25A* gene.

Sizes	Genotype frequency		Gene frequency		HWE	Ho	He	Ne	PIC
	II	0.897 (N=654)	I	0.949					
729	ID	0.103 (N=75)			P=0.340	0.902	0.098	1.108	0.093
	DD	0.000 (N=0)	D	0.051					

Note: HWE, Hardy–Weinberg equilibrium; Ho, homozygosity; He, heterozygosity; Ne, effective allele numbers; PIC, Polymorphism information content.

### 3.3 Association of the indel locus and growth-related traits of SBWC goat

**Figure 3:**
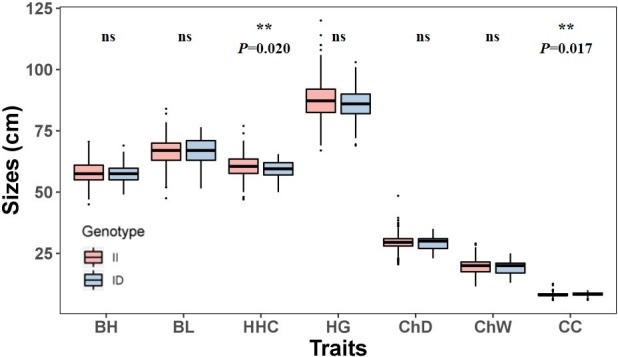
Association between the 20-bp indel locus of *CDC25A* gene and growth-related traits in SBWC goat. Note: BH, body height; BL, body length; HHC, height at hip cross; HG, heart girth; ChD, chest depth; ChW chest width; CC, cannon circumference.

The association between the 20-bp indel of *CDC25A* gene and the growth traits were investigated in the SBWC goat breeds (Tables 3, 4; Fig. 3). Significant relationships were observed between this indel locus and cannon circumference (P=0.017), and cannon circumference index (P=0.009) in SBWC goat. This indel locus also appeared to have an approximate effect on other traits such as height at hip cross (P=0.020). The height at hip cross of individuals with II genotype was higher than those with ID genotype (P=0.02); on the contrary, the cannon circumference and cannon circumference index of individuals with ID genotype were superior when compared with those with II genotype (P=0.017 and P=0.009). Besides, analysis results showed that there was no significant correlation between the other growth traits and this indel locus in SBWC goat.

**Table 3:** Relationship between the 20-bp indel locus of *CDC25A* gene and growth-related traits in SBWC goat (least square mean, LSM${}^{a}$ ± SE).

Growth traits	Genotypes		P values
	II (N=654)	ID (N=75)	
Height at hip cross (cm)	60.52±0.17	59.31±0.42	0.020
Chest width (cm)	19.62±0.12	19.20±0.33	0.255
Chest depth (cm)	29.37±0.11	29.00±0.32	0.284
Body length (cm)	66.64±0.21	66.76±0.60	0.860
Body height (cm)	57.95±0.17	57.53±0.47	0.422
Heart girth (cm)	87.17±0.29	86.17±0.85	0.264
Cannon circumference (cm)	8.14±0.03	8.34±0.08	0.017
Body trunk index (%)	131.37±0.51	129.55±1.41	0.248
Body length index (%)	115.30±0.35	116.23±0.90	0.396
Heart girth index (%)	151.04±0.59	150.36±1.74	0.713
Cannon circumference index (%)	14.09±0.05	14.53±0.14	0.009
Chest width index (%)	66.83±0.32	66.21±0.89	0.531

## 4 Discussion

*CDC25A* is specifically degraded in response to DNA damage, which prevents cells with chromosomal abnormalities from progressing through cell division. As mentioned in the literature, *CDC25A* dephosphorylates cyclin-dependent kinase and regulates the cell cycle in cell proliferation (Liang et al., 2016). In zebrafish, upon misexpression of *CDC25A*, several essential T-box transcription factors are abnormally expressed, which specifically prevents the normal onset of *myoD* transcription, leading to aberrant muscle formation (Bouldin et al., 2014). *CDC25A* can promote cell proliferation and osteoblast differentiation and ensure the health and genomic stability of the developing embryo (Verduzco et al., 2012; Qiu and Kassem, 2014). These scientific research efforts confirmed the importance of *CDC25A* in terms of growth and development. MAS is the most common method in molecular breeding. Especially indel has been widely reported in animal breeding for potential MAS (Ren et al., 2017; Cui et al., 2018; Zhao et al., 2018; Kang et al., 2019; Yang et al., 2019). Therefore, we aimed to determine the relationship between the indel polymorphism within the *CDC25A* and growth traits in goat.

Interestingly, we not only detected the II and ID genotypes, but also found a nontarget band in the *CDC25A* gene (Fig. 1). In fact, there were many similar studies about the nontarget fragment (Lu et al., 2014; Ren et al., 2017). Those nontarget bands were ultimately identified as heteroduplexes. In 1989, Nagamine et al. demonstrated that the generation of heteroduplexes theoretically occurred in any PCR reaction in which the genomic DNA carried an indel mutation. And if the heteroduplexes would be detected in the indel genes, it just existed in heterozygotes individuals (Nagamine et al., 1989). Heterozygotes in the present study showed the presence of heteroduplexes, which is consistent with the previous studies.

We firstly used an ME strategy to detect the allele frequency in all individuals (Yang et al., 2016). The present study confirmed the low frequency of this indel by randomly detecting 50 individuals one by one, and we established the most efficient pooling strategy based on the ME method. Finally, a total of 366 reaction times were performed in SBWC goat. Obviously, comparing with the one-by-one detecting method, the real times of ME-method PCR were considerably decreased. Doubtless, the successful application of ME method in our study was also consistent with previous studies (Yang et al., 2016; Li et al., 2017). Furthermore, our results found that the 20-bp indel of *CDC25A* goat gene was not in HWE in SBWC goat(P<0.05).

Furthermore, this study is the first report of the association between the 20-bp indel in the ninth intron of the *CDC25A* gene and the growth traits in SBWC goat. We found that individuals with genotype II were superior in higher height at hip cross, while they were inferior in cannon circumference and the cannon circumference index (Tables 3, 4; Fig. 3). Why? The feeding method of SBWC goats has changed from the traditional production mode of grazing to the production mode based on house feeding. Due to changes in feeding methods, some body size traits of goats have improved significantly, one of them is cannon circumference (Tan et al., 2012; Zhang et al., 2012). Therefore, we considered that goats needed bigger cannon circumference to support weight due to the needs of goat meat. As mentioned in the literature, weight gain rate of Shaanbei White Cashmere goat is relatively fast at the ages of 1 month and 4–5 months, and growth rates of body measurement indexes were relatively fast at the ages of 4–5 months and 7–9 months (Huang et al., 2017). We speculated that this discrepancy could be attributed to the lack of nutrition during development. Moreover, we did not find any individual with genotype DD in the study. We speculated that the mutation frequency of genotype DD was too low to detect.

**Table 4:** Hypothesis test summary for relationship between the genotypes from the 20-bp indel locus of *CDC25A* gene and growth traits in SBWC goat (Mann–Whitney U test).

Growth traits	Sig.	Decision${}^{*}$
Height at hip cross	0.022	reject
Chest width	0.364	retain
Chest depth	0.539	retain
Body length	0.787	retain
Body height	0.391	retain
Heart girth	0.402	retain
Cannon circumference	0.002	reject
Body trunk index	0.123	retain
Body length index	0.378	retain
Heart girth index	0.950	retain
Cannon circumference index	0.001	reject
Chest width index	0.375	retain

Note: Decision${}^{*}$: reject means that we reject the null hypothesis and retain means that we retain the null hypothesis. The null hypothesis is that the distribution of each growth trait is the same across categories of genotype. Asymptotic significance values (Sig.) are displayed. The significance level is 0.05.

In addition, although we found that this 20-bp indel was in the ninth intron of the *CDC25A* gene, the intron might also affect the phenotypic traits, which is consistent with previous reports. For example, a novel 43-bp indel polymorphism in intron 1 of the heparan sulfate 6-O-sulfotransferase 3 (*HS6ST3*) gene is significantly associated with growth and carcass traits in chickens (Wang et al., 2018b). It is also reported that a nucleotide substitution in intron 3 of *IGF2* causes a major quantitative trait loci (QTLs) effect on muscle growth in pig (Van Laere et al., 2003). Therefore, we speculated that this 20-bp indel might also affect growth traits in SBWC goats.

## 5 Conclusions

In summary, an economic ME method was presented to quickly and accurately detect a low frequency of mutation, such as the 20-bp indel in the ninth intron of the *CDC25A* gene, which can save time and reduce expenses. Besides, the detected 20-bp indel significantly affects growth traits, which might be a potential useful DNA marker for MAS in SBWC goat.

## Data Availability

The original data of the paper are available from the corresponding author upon request.
